# Utility and limitations of metabolic parameters in head and neck cancer: finding a practical segmentation method

**DOI:** 10.1007/s00405-022-07401-y

**Published:** 2022-05-02

**Authors:** Jefferson Rijo-Cedeño, Jorge Mucientes, Ithzel María Villarreal, Ana Royuela, Patricia García Vicente, José Ramón García-Berrocal

**Affiliations:** 1 Department of Otorhinolaryngology-Head and Neck Surgery, Puerta de Hierro University Hospital, Majadahonda, Spain; 2 Department of Nuclear Medicine, Puerta de Hierro University Hospital, Majadahonda, Spain; 3 Departamento de Cirugía, Universidad Autónoma de Madrid, Madrid, Spain; 4 Department of Otorhinolaryngology-Head and Neck Surgery, Fuenlabrada University Hospital, Fuenlabrada, Spain; 5 Biostatistics Unit, Biomedical Research Institute IDIPHISA, CIBERESP, Majadahonda, Spain

**Keywords:** PET, Head and neck, Cancer, MTV, TLG

## Abstract

**Purpose:**

Although metabolic tumor volume (MTV) and total lesion glycolysis (TLG) have shown good prognostic value in head and neck cancer (HNC), there are still many issues to resolve before their potential application in standard clinical practice. The purpose of this study was to compare the discrimination ability of two relevant segmentation methods in HNC and to evaluate the potential benefit of adding lymph nodes’ metabolism (LNM) to the measurements.

**Methods:**

We retrospectively analyzed a recently published database of 62 patients with HNC treated with chemoradiotherapy. MTV and TLG were measured using an absolute threshold of SUV2.5. Comparison analysis with previously published background-level threshold (BLT) results was done through Concordance index (C-index) in eight prognostic models.

**Results:**

BLT obtained better C-index values in five out of the eight models. The addition of LNM improved C-index values in six of the prognostic models.

**Conclusion:**

We found a potential benefit in adding LNM to the main tumor measurements, as well as in using a BLT for MTV segmentation compared to the most commonly used SUV2.5 threshold. Despite its limitations, this study suggests a practical and simple manner to use these parameters in standard clinical practice, aiming to help elaborate a general consensus.

## Introduction

The number of publications analyzing the prognostic value of fluorine-18-fluorodeoxyglucose (^18^F-FDG) metabolic tumor volume (MTV) and total lesion glycolysis (TLG) in head and neck squamous cell carcinoma (HNSCC) has been increasing in the last decades. Most of the studies find a good prognostic capability in these variables, as they provide valuable pretreatment information of a higher risk of recurrence [1–5]. Although widely used, maximum standardized uptake value (SUV_max_) results are more heterogeneous, and conflicting results are found in the literature regarding its prognostic potential [1, 2, 6, 7].

With the current available data, MTV and TLG seem to have a good prognostic value in HNSCC. Nevertheless, there are still several limitations to address before they can be implemented in standard clinical practice. Although most studies show good prognostic results, their methodologies vary considerably as there is no consensus yet on standardization of the calculations. First, studies differ on the volume of interest (VOI); some use the primary tumor as the VOI, and some add the metabolism of the affected lymph nodes to the primary tumor measurements. Second, studies differ on the segmentation methods, which directly affect the metabolic parameters calculations and reproducibility.

A wide variety of methods have been developed for the segmentation of VOIs in positron emission tomography (PET) scans. In general, these methods can be classified in two big groups: threshold-based and algorithm-based methods. Because of their practicality and simplicity, threshold-based methods are the most widely used in HNSCC studies. They define the burden of the VOIs based on a pre-established threshold using one of four different approaches: an absolute threshold (using all volumes above a certain SUV level [usually 2.5]), a relative threshold (using, in general, 40–50% of the SUV_max_ of the lesion), a background-level threshold (BLT) (using the SUV of a reference region, e.g., liver or mediastinum SUV), and adaptative thresholds (adjusting calculations to different measurable variables of the images, with several approaches to obtain a threshold). Algorithm-based methods constitute a heterogeneous and more complex group of methods that require specific software for segmentation of the VOIs [8, 9].

Aiming to compare two relevant segmentation methods, the purpose of this study was to evaluate the discrimination power of MTV and TLG delineated using an absolute threshold of SUV 2.5, as this method is the most used in the majority of HNSCC papers, in a combined analysis with the data of our recently reported results (where we segmented using a BLT with the liver as a reference region) [10]. Furthermore, we analyzed the potential benefit of adding the affected lymph nodes metabolism (LNM) to MTV and TLG measurements.

## Materials and methods

### Study population

We have analyzed the population published in a recent report [10]. After the approval of The Institutional Ethics Committee of our hospital, we conducted a retrospective examination of the oncological records of patients treated at Puerta de Hierro University Hospital, Majadahonda, Spain, between January 2012 and December 2018. We included patients over 18 years of age diagnosed with primary stage III–IV HNSCC without distant metastasis that had been treated with radiotherapy alone or concurrent chemoradiotherapy who underwent pretreatment 18F-FDG PET-CT for initial staging and therapy planning with no more than 6 weeks between image acquisition and treatment initiation. Exclusion criteria implicated patients with synchronous or metachronous lesions, patients who had undergone previous treatments, and patients with nasopharyngeal cancer. Informed consent was waived because of the retrospective design.

### Radiation therapy and follow-up

Patients were immobilized with a thermoplastic head-shoulders mask and simulated in supine position. Planning 3 mm slice thickness contrast-enhanced computer tomography (CT) was obtained for all patients. These images were combined with pretreatment 18F-FDG PET for better delineation of treatment volumes. Clinical target volumes (CTV) were as follows: CTV1: primary and affected lymph nodes gross tumor volumes; CTV2: CTV1 and the “high risk” first uninvolved lymph node region; CTV3: elective bilateral lymph nodes concurring with international guidelines [11]. By adding 0.5 mm to the CTVs, we generated the corresponding planning target volumes (PTVs). Treatment with intensity modulated radiation therapy was administered in a TomoTherapy HiArt unit equipped with an image-guided radiation therapy system and was dispensed in 32 fractions using the simultaneous integrated boost-up to doses for PTV1, PTV2, and PTV3 of 69.12, 57.6 and 53.12 Gray (Gy), respectively. Dose-limiting constraints for organs at risk were the following: Dmax of 45 Gy for spinal cord; V28 < 50% for parotid glands; V65 < 10% for mandible; Dmax of 55 Gy for brain stem; and Dmean < 50 Gy for constrictor muscles.

Two protocols consisting in a regimen of cisplatin (two courses of 20 mg/m2/d in days 1–4 and 29–32) plus oral tegafur (two courses of 1200 mg/d in days 1–14 and 29–43) [12], and a regimen of six cycles of weekly cisplatin (40 mg/m^2^) were administered as concomitant chemotherapy for most patients. Patients who were not fit for a cisplatin-based protocol were treated with Cetuximab (400 mg/m^2^ initial dose, followed by seven weekly doses of 250 mg/m^2^). Patients whose physical conditions were not suitable for chemotherapy were treated with radiotherapy alone.

Two to four months after treatment completion, an ^18^F-FDG PET-CT was performed in all patients to assess adequate treatment response. Follow-up was done every 6 months for the first 3 years and once a year for 2 more years with head and neck imaging (CT or magnetic resonance imaging). Salvage surgery or palliative treatment was indicated in case or recurrent or persisting disease.

### ^18^F-FDG PET-CT imaging

Prior to the ^18^F-FDG i.v. injection (approximately 350–400 MBq), patients were required to have a serum glucose concentration bellow 180 mg/dL after fasting for at least 6 h. A helical multidetector CT was used to perform non-contrast-enhanced scans from the top of the skull to the mid-tight with the following parameters: 110 kVp, a maximum modulated milliamperage of 85 mAs, and six slices with a 5.0 mm thickness. CT images were then used for image fusion and attenuation correction. A multimodality Siemens Biograph 6 scan (Biograph; Siemens, Erlangen, Germany) was used to acquire the PET images after 50–60 min of ^18^F-FDG i.v. injection with the following parameters: 4-min’ scan per bed position × 7–8 positions and ordered-subset expectation maximization reconstruction (four iterations, eight subsets).

### Image interpretation

^18^F-FDG PET images were analyzed by an experienced nuclear medicine practitioner (J.M.) using a Siemens Leonardo reading station. Patient’s clinical outcomes were unknown during the analysis using the program Syngo®.via (Siemens Healthineers, Muenchen, Germany). To obtain semiquantitative metabolic parameters, image pixels were converted into standardized uptake values. Then, we set a SUV of 2.5 for VOI segmentation of the tumor and the affected lymph nodes. All ^18^F-FDG-avid lesions were then manually encircled in three imaging planes, and the volumes with ≥ SUV2.5 were defined as the VOIs. Tumor and lymph nodes’ MTV were automatically calculated by the software, and those two volumes were then analyzed individually and combined. TLG was obtained using the formula: MTV x SUVmean.

### Statistical analysis

First, we made a descriptive analysis representing categorical variables with absolute and relative frequencies. Numerical variables were evaluated through medians with percentiles 25 and 75 (P25-P75), means with standard deviation, and minimum and maximum values.

For the survival analysis, overall survival (OS) was defined as the time between treatment initiation and death from any cause. Disease-free survival (DFS) was defined as the time between treatment initiation and relapse or death from any cause, whichever happened first. Patients who were followed-up until the end of the study with no events, as well as patients lost in the follow-up with no events were censored at the date of last contact. Kaplan–Meier curves were estimated and differences between survival curves were compared through the log-rank test. Survival analysis was made with the combination of lymph nodes and main tumor MTV and TLG.

To obtain the optimal cutoff points, receiver-operating characteristics (ROC) curves with the survival data were developed [13], and the points were also evaluated with the Youden [14] and Liu [15] methods. To assess the association between MTV and TLG measured by SUV2.5 with OS and DFS, Cox proportional hazard models were executed. We tested each variable in two ways: as a continuous variable (per 10-ml increment for MTV and per 25-g increment for TLG) and as a dichotomized variable with the obtained cutoff points. We then developed an univariable approach, and a multivariable model adjusting by age, T-classification, and N-classification. Proportional hazards assumption was checked using Schoenfeld residuals. Hazard ratios (HR) with their corresponding 95% confidence intervals are shown.

To test the discrimination ability of the models, Harrell’s/Concordance Index (C-index) was estimated. Values of C-index near 0.5 indicate that the discrimination ability is no better than chance. Values near 1 indicate that the model is good at determining which of two patients will have an event first [16].

To evaluate the effect of lymph nodes’ metabolism in the discrimination ability of the variables, we created several univariable regression models for tumor, lymph nodes’, and tumor + lymph nodes’ MTV and TLG with both absolute threshold method (SUV2.5) and our previous published data with a background-level threshold method. [10]. To compare the latter two segmentation methods, we analyzed eight prognostic models using the combined data from both studies: MTV and TLG continuous and dichotomized models with the two segmentation methods mentioned before.

Significance level was set at 0.05. Software used was Stata 16 (StataCorp. 2019. *Stata Statistical Software: Release 16*. College Station, TX: StataCorp LLC.)

## Results

### Demographics

Seventy-nine oncological records were assessed from patients diagnosed with stage III and IV HNSCC. Two patients had a longer than 6 weeks interval between treatment initiation and PET acquisition and thus were excluded. Pre-treatment ^18^F-FDG PET-TC was performed in a different center in 15 patients and were also excluded. Therefore, 62 records were fully assessed for the analysis. The median age was 65 years (range, 38–87 years) and the female/male distribution was 13/49. Forty-nine patients were smokers. Disease locations were: oral cavity (OC): 7 patients, hypopharynx (HP): 6 patients, oropharynx (OP): 16 patients (7 human papilloma virus [HPV] positive, 6 HPV negative, and not stated HPV status in 3 patients), and larynx (L): 33 patients.

T-classification findings were as follows: T1: 2 patients; T2: 16 patients; T3: 29 patients; and T4: 15 patients. N-classification included: N0: 14 patients; N1: 5 patients; N2: 37 patients; and N3: 6 patients. Twenty-two patients had a AJCC stage III disease, 29 had a stage IVa, and 11 had a stage IVb. Most patients received treatment with concomitant chemoradiotherapy. Thirty-one of them were treated with a chemotherapy protocol of cisplatin + tegafur (OC: 4, OP: 6, HP: 4, and L: 17), 17 with a weekly cisplatin protocol (OC: 1, OP: 5, HP: 1, and L: 10), 11 with a cetuximab protocol (OC: 2, OP: 2, HP: 1, and L: 6), and 3 patients were treated with radiotherapy alone (OP: 3). Two patients died during treatment and, because of a severe case of radiodermatitis, one patient refused to complete the treatment.

### PET parameters’ determination

Respectively, the median values for TLG and MTV were 176.92 g (range 5.5–2,567.04 g) and 29.94 ml (range 1.66–220.87 ml). Cutoff values estimated at a 3-years’ time point for TLG and MTV were acquired using a time-dependent AUC-ROC. The same values were obtained using the Youden and Liu methods. The cutoff points were 284 g for TLG (AUC = 0.68) and 60 ml for MTV (AUC = 0.70). Subsequently, Cox regression analyses were performed dichotomizing the corresponding cutoff values.

### Survival analysis

The median follow-up was 59.3 months (95% CI; 48.6–68.5 months). Locoregional recurrence occurred in 21 patients and distant metastasis was found in 13 patients. Four-year DFS was 57% (95% CI 43–69). Four-year overall survival (OS) was 63% (95% CI 49–74). Global 4-year OS and DFS per disease location was, respectively, as follows: OC: 50% (95% CI = 11–80) and 65% (95% CI = 35–84); OP: 65% (95% CI = 35–44) and 48% (95% CI = 11–80); HP: 50% (95% CI = 11–80) and 20% (95% CI = 05–75); L: 68% (95% CI = 48–81) and 60% (95% CI = 41–75).

OS and DFS of larynx patients per stage were, respectively, as follows: Stage III, 75 (95% CI = 40–91) and 73 (95% CI = 37–90); Stage IVa, 64 (95% CI = 35–82) and 54 (95% CI = 28–74); Stage IVb, 50 (95% CI = 06–91) and 50 (95% CI = 0.6–91). OS and DFS of oropharynx patients per stage were, respectively, as follows: Stage IVa, 48 (95% CI = 0.7–81) and 38 (95% CI = 06–71); Stage IVb, 75 (95% CI = 12–96) and 75 (95% CI = 12–96). It was only possible to estimate the overall survival for patients with stage III OP cancer with an OS of 80 (95% CI = 20–97). OS and DFS of oral cavity patients in stage IVb were 25 (95% CI = 0.8–67) and 25 (95% CI = 0.8–67). OS and DFS of hypopharynx patients in stage III were 33 (95% CI = 0.9–77) and 33 (95% CI = 0.9–77). It was not possible to estimate survival in stages III nor IVa OC patients nor in stages IVa and IVb of HP patients because of the absence of events during the follow-up. DFS of stage III OP cancer was not estimable as well.

Primary local control was achieved in 12 (75%), 4 (57%) 3 (50%) and 24 (73%) of the patients with OP, OC, HP, and L disease, respectively. Primary local–regional control was achieved in 11 (69%), 4 (57%), 3 (50%), and 23 (70%) of the patients with OP, OC, HP, and L disease, respectively. A broader detailed description of the study population is described in our previous report [10].

There was a significant association with OS in both dichotomized [hazard ratio (HR) = 2.61, 95% CI = 1.18–5.81, *p* = 0.018] and continuous (HR = 1.11, 95% CI = 1.04–1.19, *p* = 0.002) MTV models in the univariable analysis. Dichotomized (HR = 2.48, 95% CI = 1.13–5.45, *p* = 0.024) and continuous (HR = 1.03, 95% CI = 1.01–1.05, *p* = 0.001) TLG models also had a significant association with OS. There was also a significant association with DFS in the dichotomized (HR = 3.06, 95% CI = 1.38–6.76, *p* = 0.006) and continuous (HR = 1.16, 95% CI = 1.08–1.25, *p* < 0.001) MTV models, as well as in the dichotomized (HR = 2.87, 95% CI = 1.23–6.35, *p* = 0.009) and continuous (HR = 1.03, 95% CI = 1.01–1.05, *p* < 0.001) TLG models in the univariable analysis (Table 1).

**Table 1:** Cox regression analyses for MTV and TLG segmented with SUV2.5

	Overall survival				Disease-free survival			
	Univariable analysis		Multivariable analysisª		Univariable analysis		Multivariable analysis†	
	HR (95% CI)	*p*-value	HR (95% CI)	*p*-value	HR (95% CI)	*p*-value	HR (95% CI)	*p*-value
Dichotomized MTV model		0.018		0.010		0.006		0.007
≤ 60 ml	1		1		1		1	
> 60 ml	2.61 (1.18–5.81)		3.06 (1.31–7.12)		3.06 (1.38–6.76)		3.07 (1.36–6.94)	
Continious MTV model		0.002		0.002		< 0.001		< 0.001
Per 10-ml increment	1.11 (1.04–1.19)		1.12 (1.04–1.22)		1.16 (1.08–1.25)		1.16 (1.71–1.26)	
Dichotomized TLG model		0.024		0.013		0.009		0.011
≤ 284 g	1		1		1		1	
> 284 g	2.48 (1.13–5.45)		2.86 (1.25–6.48)		2.87 (1.23–6.35)		2.91 (1.28–6.64)	
Continious TLG model		0.001		0.001		< 0.001		0.001
Per 25-g increment	1.03 (1.01–1.05)		1.03 (1.01–1.05)		1.03 (1.01–1.05)		1.03 (1.01–1.05)	

ªAdjusted by age, T-classification, and N-classification

*HR* hazard ratio, *CI* confidence interval, *MTV* metabolic tumor volume, *TLG* total lesion glycolysis, *C-index* Harrell’s C-index, *ml* milliliter, *g* gram

Both MTV and TLG models remained as independent prognostic factors for DFS and OS in the multivariable analyses. Patients with a > 284 g TLG had a higher hazard for recurrence (HR = 2.91, 95% CI = 1.28–6.64, *p* = 0.011) and death (HR = 2.86, 95% CI = 1.25–6.48, *p* = 0.013). The HR for every 25-g increment of TLG was 1.03 (95% CI = 1.01–1.05, *p* = 0.001) for DFS, and 1.03 (95% CI = 1.01–1.05, *p* = 0.001) for OS. Patients with > 60 ml MTV also had a higher hazard for recurrence (HR = 3.07, 95% CI = 1.36–6.94, *p* = 0.007) and death (HR = 3.06, 95% CI = 1.31–7.12, *p* = 0.010). HR for every 10-ml increment of MTV was 1.16 (95% CI = 1.71–1.26, *p* =  < 0.001) for DFS, and 1.12 (95% CI = 1.04–1.22, *p* = 0.002) for OS (Table 1).

**Fig. 1:**
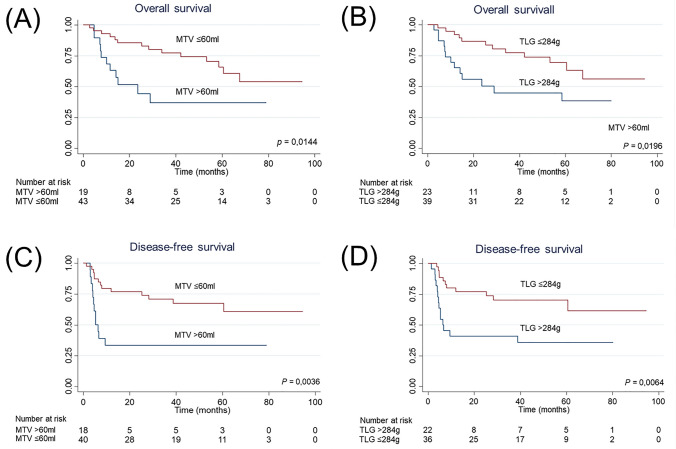
Kaplan–Meier’s curves for DFS and OS using the MTV and TLG dichotomized models. A, Curves for OS and a cutoff of 60 ml of MTV. B, Curves for OS and a cutoff of 284 g of TLG; C, curves for DFS and a cutoff of 60 ml of MTV. D, Curves for DFS and a cutoff of 284 g of TLG. *MTV* metabolic tumor volume, *TLG* total lesion glycolysis, *ml* milliliter, *g* gram

Kaplan–Meier’s curves for DSF and OS using the dichotomized models of MTV and TLG are shown in Fig. 1.

### Comparison analysis

Univariable analyses per unit increment of lymph nodes’, tumor, and the combination of tumor and lymph nodes’ MTV found a significant association with OS and DFS in both background-level and absolute SUV2.5 segmentation methods (Table 2). The same happened with TLG, except for tumor TLG measured with the BLT method, which did not have a significant association with OS nor DFS (Table 3). The determination of tumor + lymph nodes’ TLG obtained better C-index values than the determination of tumor TLG alone with both segmentation methods. The same happened with MTV measurements using the absolute SUV2.5 method. On the contrary, C-index values were lower with the addition of LNM to tumor MTV measurements with the background-level method for both OS and DFS (Tables 2–3).

**Table 2:** Univariable Cox regression analyses for the volumes of interest of MTV

	Overall survival			Disease-free survival		
	HR (95% CI)ª	*p*-value	C-index	HR (95% CI)ª	*p*-value	C-index
T MTV with SUV2.5 threshold	1.02 (1.01–1.03)	0.009	0.656	1.02 (1.01–1.03)	0.002	0.695
N MTV with SUV2.5 threshold	1.01 (1.003–1.027)	0.013	0.586	1.01 (1.003–1.02)	0.012	0.587
T + N with MTV SUV2.5 threshold	1.01 (1.01–1.02)	0.002	0.667	1.01 (1.01–1.02)	< 0.001	0.703
T MTV with background-level threshold	1.02 (1.01–1.04)	0.005	0.659	1.03 (1.01–1.0)	0.001	0.688
N MTV with background-level threshold	1.02 (1.004–1.04)	0.011	0.575	1.01 (1.002–1.03)	0.025	0.574
T + N MTV with background-level threshold	1.02 (1.01–1.03)	0.002	0.656	1.02 (1.01–1.03)	< 0.001	0.681

ªPer unit increment

*HR* hazard ratio, *CI* confidence interval, *MTV* metabolic tumor volume, *C-index* Harrell’s C-index, *SUV* standardized uptake value, *T* tumor, *N* lymph node

**Table 3:** Univariable Cox regression analyses for the volumes of interest of TLG

	Overall survival			Disease-free survival		
	HR (95% CI)ª	*p*-value	C-index	HR (95% CI)ª	*p*-value	C-index
T TLG with SUV2.5 threshold	1.001 (10,004–1.003)	0.009	0.652	1.003 (1.001–1.004)	0.001	0.693
N TLG with SUV2.5 threshold	1.002 (1.001–1.003)	0.005	0.586	1.001 (1.001–1.002)	0.014	0.586
T + N with TLG SUV2.5 threshold	1.001 (1.001–1.002)	0.001	0.660	1.001 (1.001–1.002)	< 0.001	0.706
T TLG with background-level threshold	1.001 (1.01–1.001)	0.281	0.629	1.001 (1.01–1.001)	0.245	0.671
N TLG with background-level threshold	1.002 (1.001–1.003)	0.006	0.545	1.001 (1.001–1.002)	0.020	0.566
T + N TLG with background-level threshold	1.001 (1.001–1.002)	0.001	0.649	1.001 (1.001–1.002)	< 0.001	0.694

ªPer unit increment

*HR* hazard ratio, *CI* confidence interval, *TLG* total lesion glycolysis, *C-index* Harrell’s C-index, *SUV* standardized uptake value, *T* tumor, *N* lymph node

Among the eight models, we created comparing the two segmentation methods, BLT had a better C-index in five of them. The four dichotomized models performed better with the BLT method. When comparing the continuous models, SUV 2.5 models worked better to predict the DFS. When predicting OS, only the continuous model of SUV 2.5 MTV had better C-index values than the BLT (Table 4).

**Table 4:** C-index comparison analysis with multivariable models of the analyzed segmentation methods

Model	C-index values for overall survival		C-index values for disease-free survival	
	Absolute threshold of SUV2.5	Background-level thresholdª	Absolute threshold of SUV2.5	Background-level thresholdª
Dichotomized MTV model	0.664	0.673	0.657	0.673
Continuous MTV model	0.681	0.676	0.677	0.676
Dichotomized TLG model	0.676	0.696	0.669	0.675
Continuous TLG model	0.662	0.663	0.678	0.677

ªUsing the liver as the reference region

*MTV* metabolic tumor volume, *TLG* total lesion glycolysis, *C-index* Concordance index, *ml* milliliter, *g* gram

## Discussion

Although MTV and TLG have consistently shown good prognostic value in patients with HNSCC in many studies [1–5], there are still several factors limiting their application in standard clinical practice. Because of the variability in the methodology used in the different publications, it is not possible to stablish adequate nor generalizable cutoff values. For their correct implementation, it is necessary to firstly standardize one segmentation method among the many available ones, as well as establishing whether we should incorporate the metabolism of the affected lymph nodes in the measurements or not.

In this study, we evaluated the prognostic value of MTV and TLG delineated using an absolute threshold of SUV 2.5 in our recently reported database (where we segmented using a BLT with the liver as a reference region) [10]. Subsequently, we created eight prognostic models using the results of both studies to assess the discrimination power of the two methods through their C-index values. Our results found an independent statistical association with survival for both methods in this population (Table 1), and the BLT obtained better C-Index values in five out of the eight prognostic models, including all dichotomized models (Table 4).

We have found several studies comparing threshold and algorithms’ segmentation methods, and the latter ones seem to perform better at discriminating patients at risk of recurrence [17–22]. It is to be expected that more complex and specific methods would provide a better prognosis prediction. However, considering the many different algorithms available and the limited studies found from each of them, it is difficult to imagine any of these methods having a widespread application at the moment. The same happens with adaptative thresholds, where there is no consensus yet on which factors to consider for the calculations [8]. Thus, threshold methods seem to be the most practical and reproducible segmentation methods with current PET scanners, and they have systematically provided good prognostic information in HNSCC.

Among the five studies comparing fixed absolute, fixed relative, and background-level threshold methods we have found, four of them obtained a better result with the latter, concurring with our results [19, 23–25]. One study did not find a statistical significance result for the background-level method [26]. Absolute and relative threshold methods are dependent of the standardized uptake value (SUV) and, therefore, are directly affected by SUV variations. Patients’ preparation and characteristics, PET scanner quality and differences in image analysis protocols have a direct impact in SUV calculations [27–29]. To reduce this variability, some authors suggest to either use a background-level threshold [24, 28, 30, 31] or a gradient-based method [18, 20, 21].

To analyze the potential benefit of adding lymph node’s metabolism to the measurements, we created eight prognostic models in a combined analysis (Tables 2–3). We found an association between tumor and lymph nodes’ MTV and TLG with survival in six of them. Only tumor TLG using the BLT method did not have a statistical significance for neither DFS nor OS. After the addition of the LNM to the main tumor measurements, we obtained better C-index values in six of the models, and BLT tumor TLG gained statistical significance for DFS and OS. Only tumor MTV using the BLT method got lower values of C-index after the addition of the LNM. These results exhibit a possible prognostic benefit in the combination of both measurements.

In the literature, besides Castelli et al. [32] and this study, most papers do not find a statistical association between LNM and survival in HNSCC [33–40], although it may play a role in patients with HPV + oropharyngeal cancer [41–44] and in hypopharyngeal patients with a low MTV [35]. Hoshikawa et al. [45] share the same findings we obtained in our BLT tumor TLG models, where statistical significance was achieved only after the addition of the LNM. To the best of our knowledge, this is the first study testing the possible benefit of combining both tumor and lymph node’s measurements in HNSCC.

This study has numerous limitations. The potential biases of a retrospective data compilation in a reduced single-center population with different disease locations limit the generalization of the results. Although there was a tendency of a better performance of the BLT method, the difference in the C-index values were millimetric. Even so, and with all due limitations, this study addresses major concerns in the implementation of MTV and TLG in today’s clinical practice, hoping to help elaborate a consensus on how to measure these variables.

Although PET’s metabolic parameters seem to offer valuable prognostic information and, perhaps in the future, could aid in treatment selection [46] and in dynamic radiation dose modification [47], more prospective and multicentric studies with rigorous standardization in PET preparation are still needed to obtain quality evidence regarding how and when to use these parameters in HNSCC. Our results found a possible statistical benefit in adding lymph nodes metabolism to the measurements, as well as in using a BLT for segmentation compared to the most used SUV2.5 threshold. Considering this method only adds no more than a minute of work and can be measured with current PET scanners with no specific software, it may be a suitable choice to evaluate MTV and TLG in HNSCC patients.

## Conclusions

Out of the eight prognostic models created, the BLT method had a better prediction power than SUV2.5 in five of them, and the addition of the affected lymph node’s metabolism to MTV and TLG measurements improved their prediction power in six of the eight models. Despite the limitations of this study, our results suggest a practical and simple manner on which these metabolic parameters can be implemented in today’s clinical practice.
